# Supplementary material for: A sparse code for natural sound context in auditory cortex
Source: Curr Res Neurobiol. 2023 Nov 29;6:100118. doi: 10.1016/j.crneur.2023.100118 (PMC10749876; doi:10.1016/j.crneur.2023.100118)
Supplement: Multimedia component 1 [file mmc1.pdf]

A sparse code for natural sound context in auditory cortex

Mateo López Espejo[1], Stephen V. David[2]

[1]Neuroscience Graduate Program, [2]Otolaryngology, Oregon Health & Science University, Portland, Oregon, USA.

Corresponding author: SVD, davids@ohsu.edu

Supplementary Figures

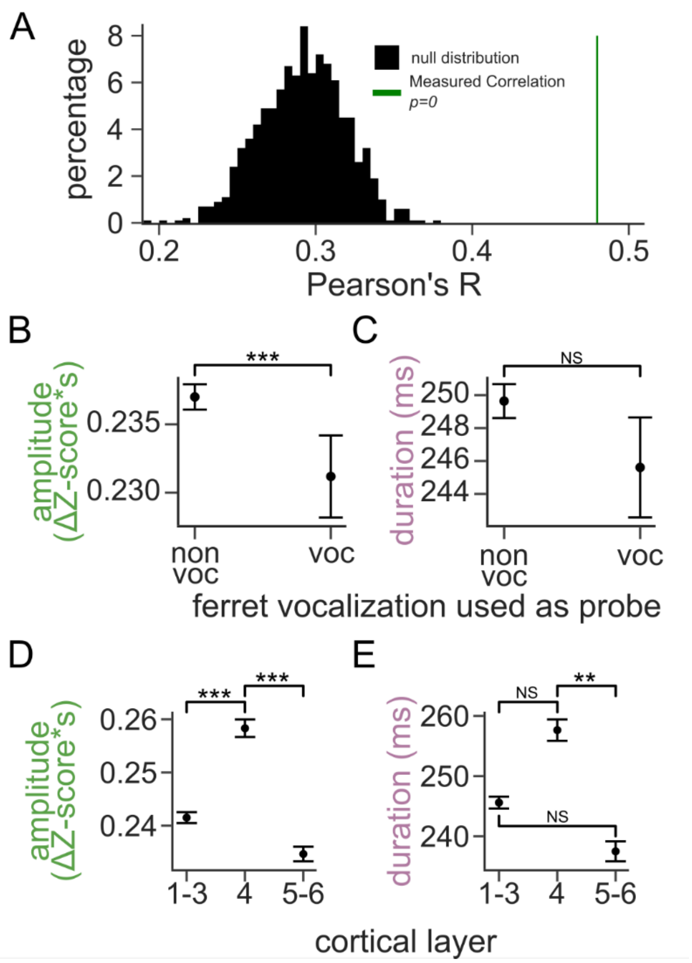

Supplementary Figure 1. Context metrics correlation, and diminished context effects with species-specific vocalization probe stimuli. A. Null distribution of the correlation between amplitude and duration context metrics, obtained by shuffling context effects over time (black histogram, mean ± SEM: 0.29 ± 0.0008), and the measured correlation (green line, R = 0.479). **B.** Amplitude of context effects on a probe that was either a ferret vocalization or not (mean ± SEM: *non-vocalization*: 0.24 ± 0.0009, *vocalization*: 0.23 ± 0.0030 Z-score*s. Kruskal Wallis $p < 0.001$). **C.** Same as in A for context effect duration (mean ± SEM: *non-vocalization*: 249.64 ± 1.0**3**, *vocalization*: 245.61 ± 3.0**4** ms. Kruskal Wallis $p = 0.925$). **D.** Context effect amplitude as a function of cortical layer (mean ± SEM: 1-3: 0.241 ± 0.001, 4: 0.258 ± 0.001, 5: 0.234 ± 0.001, Z-score*s. Kruskal Wallis $p < 0.001$, Dunn post hoc test with Bonferroni correction, $p < 0.001$ for all comparisons). **E.** same as in D but for duration (mean ± SEM: 1-3: 245.5 ± 0.99, 4: 257.6 ± 1.76, 5: 237.5 ± 1.66, ms. Kruskal Wallis $p = 0.011$, Dunn post hoc test with Bonferroni corrections, $p < 0.01$ between L4 and L5-6).

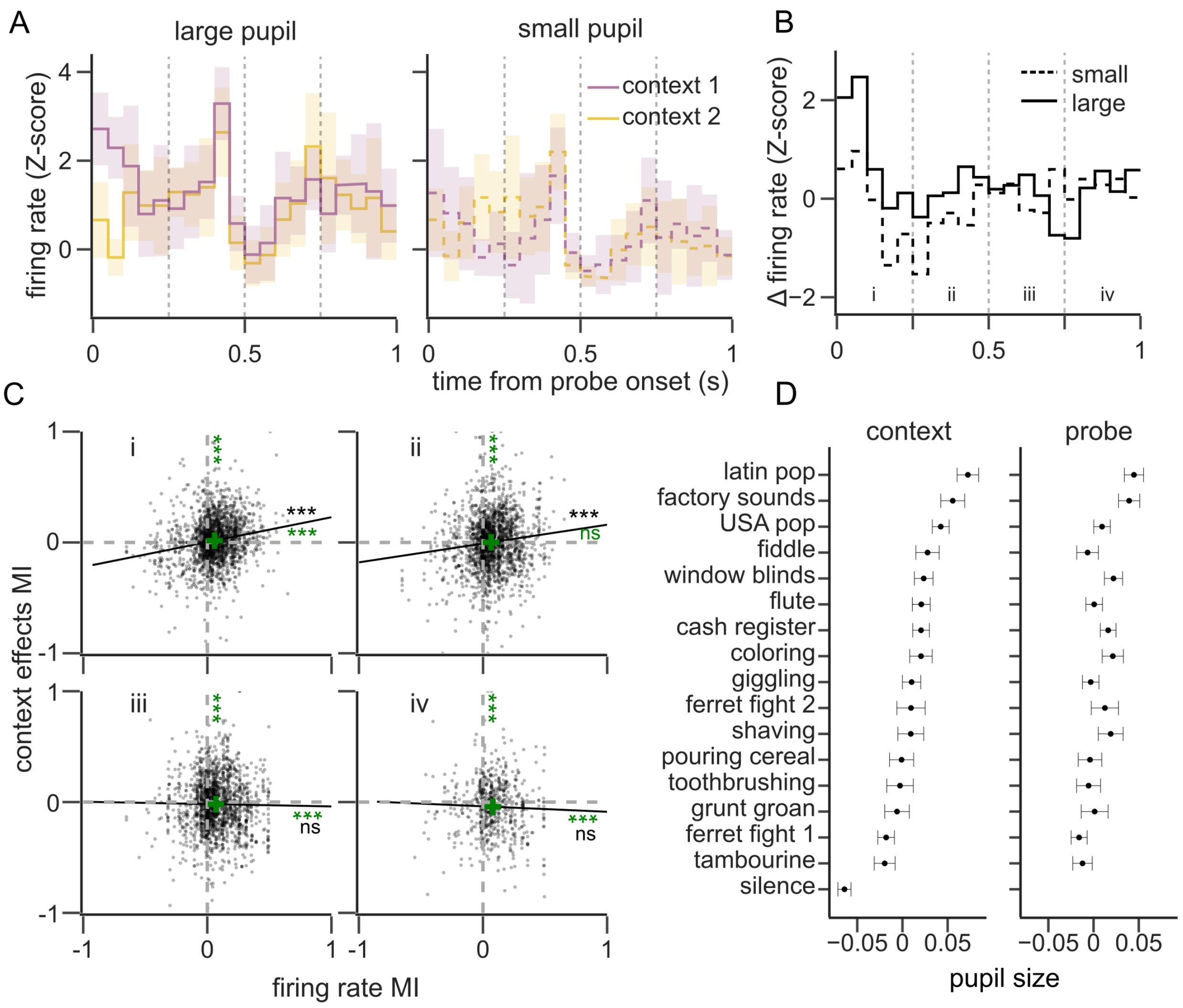

Supplementary Figure 2. Large pupil is weakly correlated with decreased context effects. A. Example PSTH response of one neuron (line: mean, shade: SEM) to a probe sound following two contexts (color) for trials with large (left, solid lines) and small (right. dashed lines) pupil. When pupil is small, an overall reduction in firing rate reduces the amplitude of early context effects. **B.** Context-dependent difference (difference between context 1 and 2) for large (solid line) and small (dashed lines) pupil trials of the example in A. **C.** Comparison between pupil modulation index (MI: -1 to +1 for greater effects with small or large pupil, respectively) of firing rate (MI-fr) and context effect amplitude (MI-ce) for individual instances during 250ms intervals labeled in panel B (i to iv, n = 12868, 4942, 1815, 668, dots decimated for clarity). Vertical and horizontal dashed gray lines indicate MI=0 (no pupil effect). Green crosses indicate means on x and y. Significance of mean difference from zero on x and y is indicated with green

symbols on the top and right sides (one sample T-test). Black lines indicate linear regression, with associated significance indicated in black (Wald test for nonzero slope). On average, firing rate was increased with large pupil during all intervals (mean MI-fr: i: 0.057; ii: 0.065; iii: 0.069; iv: 0.079. $p<0.001$ for all time intervals), while large pupil was associated with greater context effects during earlier intervals and smaller context effects during later intervals (mean MI-ce, p-value: i: 0.016, <0.0; ii: 0.001, 0.7; iii: -0.019, <0.001; iv: -0.042, <0.001). Dependence of firing rate and context effects on pupil size were significantly correlated during early intervals but not late intervals (Pearson's R, p-value: i: 0.17, <0.001; ii: 0.11, <0.001; iii: -0.01, 0.5; iv: -0.03, 0.3). Statistical significance symbols, ***: $p<0.01$, ns: non-significant. **D.** effect of different sounds as context (left) or probe (right) on the mean pupil amplitude during the probe interval. Markers and whiskers indicate the mean and SEM. Significance was tested with Kruskal Wallis (context: statistic = 155.49, $p < 0.001$; probe: statistic = 43.39, $p < 0.001$), and Dunn post hoc tests with Bonferroni corrections, which shows significant differences between pairs of sounds, e.g., *silence* and all other sounds used as context ($p < 0.05$ up to *shaving*, $p < 0.001$ past *shaving*) and *latin pop* significantly different than some sound used as context ($p < 0.01$ past *giggling.* Significance symbols are omitted as they are too numerous to show).

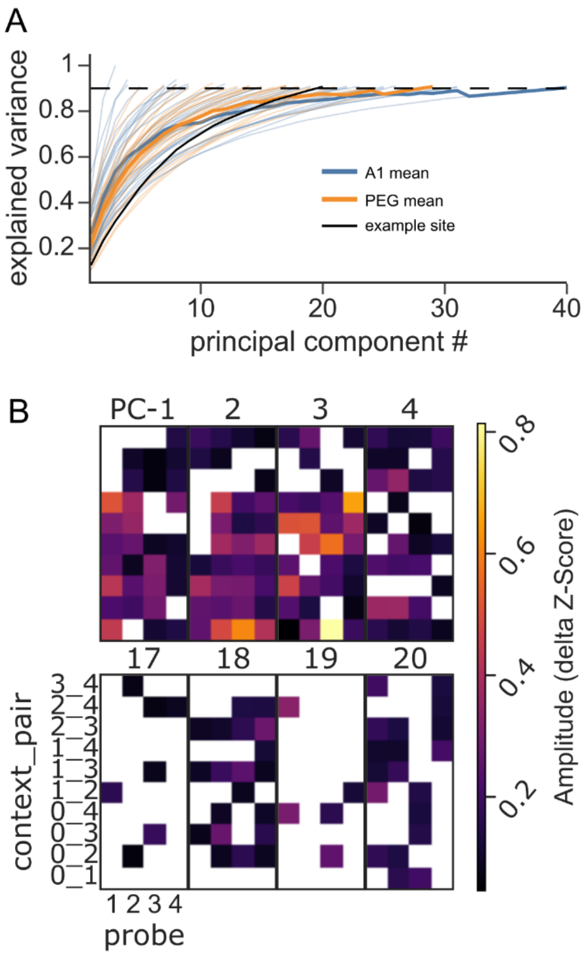

Supplementary Figure 3. Principal Components capture a small fraction of context effects. A. Fraction of variance explained by principal components (PCs) for all recorded sites in A1 (blue) and PEG (orange). Individual sites and average are indicated by faint and bold lines respectively. The black line indicates the example site used in Fig. 3 and panel B. PCs were calculated up to 90% variance explained (horizontal dashed line). **B.** Context coverage of the 4 first and last PC, shown as, and using the same example site of Fig. 3A.

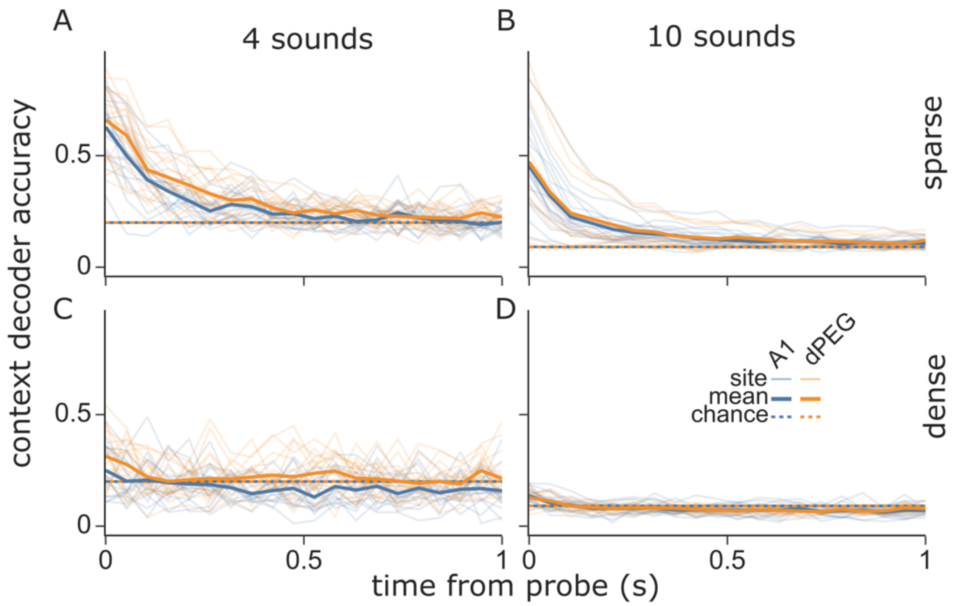

Supplementary Figure 4. Decoding accuracy for different cortex regions and sound sets. A. Support vector machine accuracy at decoding the context identity from the population activity of sites (thin lines) and their mean (thick lines), recorded at A1 (blue) and dPEG (orange), subject to stimuli composed of 4 different sounds (5 contexts, including silence). Chance performance is indicated with dotted lines. **B.** Same as in A but for stimuli composed of 10 different sounds (11 contexts). **C-D.** Same as in A-B but after transforming the data to impose a dense representation of the context information.

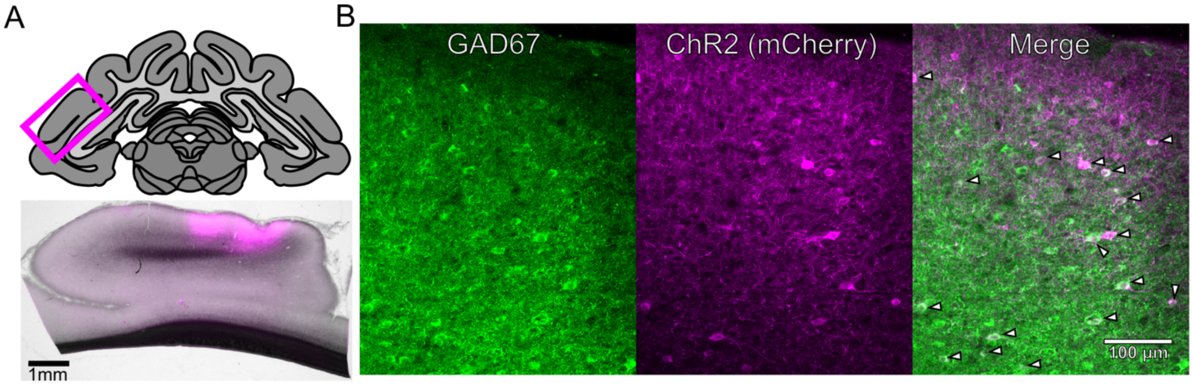

Supplementary Figure 5. Validations of Channelrhodopsin-2 expression in inhibitory interneurons. A. Top: schematic of a coronal section of the ferret brain encompassing a section of the ectosylvian gyrus and A1, indicated by the magenta box. Bottom: bright field image with superimposed red fluorescence of the virally transduced cell (mCherry, magenta) around the injection sites on the ectosylvian gyrus indicated above. Scale bar: 1mm. **B.** immunohistochemistry of the injection site in A, showing GAD67 positive inhibitory interneurons (green), transduced cells expressing ChR2 alongside the reporter mCherry, under the inhibitory interneuron specific promoter mDlx (magenta), and the channel merge with arrows indicating neurons co-expressing the markers. Most transduced neurons are inhibitory interneurons. Scale bar: 100 µm.

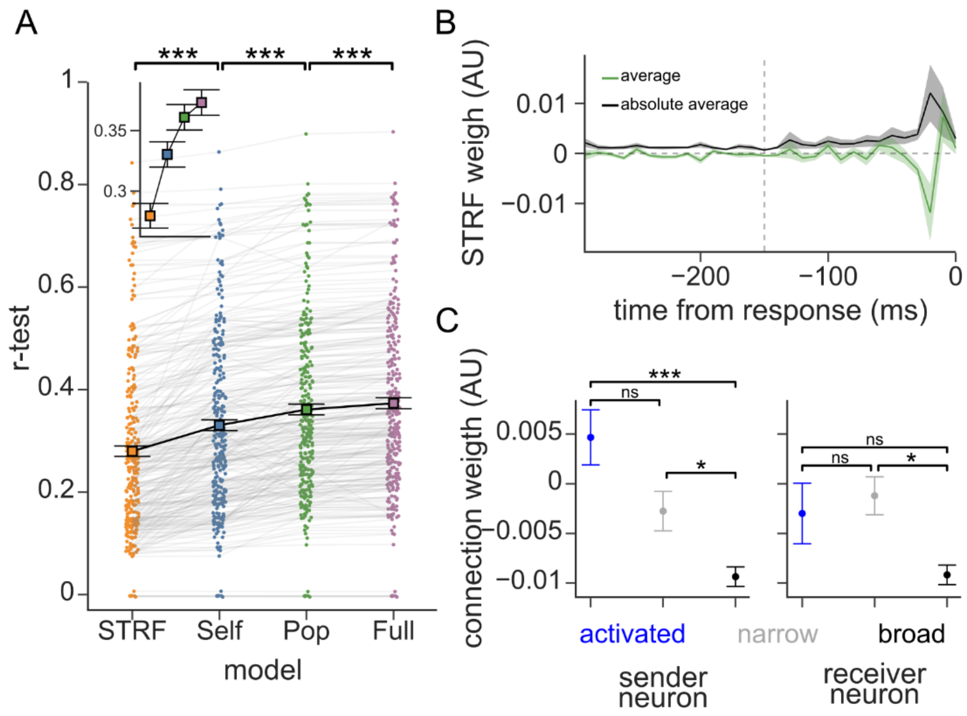

Supplementary Figure 6. Absolute model performance and weights summary. A. Quantification of prediction accuracy (cross validated Pearson's r) for each model. Colored circles connected by gray lines represent models fit to individual neurons (n=275). Black squares and error bars are the mean and SEM with a corresponding trend line. Inset shows the mean and SEM alone for clarity (Wilcoxon signed-rank test, Bonferroni corrected, same as in Fig. 6C). **B.** Full model STRF weight over time prior to model prediction. Weights were averaged across spectral channels (green). To prevent weights with different sign cancelling out, the absolute value las considered (black). Lines and shades represent the mean and SEM for all fitted neurons (n=314). **C.** Full model population connection weights for neurons classified by optotagging (blue) and spike waveform (black and gray). The same weights are classified twice by the sending (left) and receiving (right) neuron type in the connection. Markers and whiskers indicate mean and SEM (ns: non-significance, *: $p < 0.05$, **: $p < 0.01$, ***: $p < 0.001$, Kruskal Wallis with Dunn post hoc test with Bonferroni corrections).
